# Establishment of Adenomyosis Organoids as a Preclinical Model to Study Infertility

**DOI:** 10.3390/jpm12020219

**Published:** 2022-02-04

**Authors:** Elena Juárez-Barber, Emilio Francés-Herrero, Ana Corachán, Carmina Vidal, Juan Giles, Pilar Alamá, Amparo Faus, Antonio Pellicer, Irene Cervelló, Hortensia Ferrero

**Affiliations:** 1 Fundación IVI, Instituto de Investigación Sanitaria La Fe, 46026 Valencia, Spain; elena.juarez@ivirma.com (E.J.-B.); emilio.frances@ivirma.com (E.F.-H.); amparo.faus@ivirma.com (A.F.); apellicer@ivirma.com (A.P.); irene.cervello@ivirma.com (I.C.); hortensia.ferrero@ivirma.com (H.F.); 2 Departamento de Pediatría, Obstetricia y Ginecología, Universidad de Valencia, 46010 Valencia, Spain; 3 Departament of Gynecology, IVIRMA-Valencia, 46015 Valencia, Spain; carmina.vidal@ivirma.com (C.V.); juan.giles@ivirma.com (J.G.); pilar.alama@ivirma.com (P.A.); 4 Departament of Gynecology, IVIRMA-Rome, 00197 Rome, Italy

**Keywords:** adenomyosis, organoids, secretory endometrium, gestational endometrium, preclinical model, infertility

## Abstract

Adenomyosis is related to infertility and miscarriages, but so far there are no robust in vitro models that reproduce its pathological features to study the molecular mechanisms involved in this disease. Endometrial organoids are in vitro 3D models that recapitulate the native microenvironment and reproduce tissue characteristics that would allow the study of adenomyosis pathogenesis and related infertility disorders. In our study, human endometrial biopsies from adenomyosis (*n* = 6) and healthy women (*n* = 6) were recruited. Organoids were established and hormonally differentiated to recapitulate midsecretory and gestational endometrial phases. Physiological and pathological characteristics were evaluated by immunohistochemistry, immunofluorescence, qRT-PCR, and ELISA. Secretory and gestational organoids recapitulated in vivo glandular epithelial phenotype (pan-cytokeratin, Muc-1, PAS, Laminin, and Ki67) and secretory and gestational features (α-tubulin, SOX9, *SPP1*, *PAEP*, *LIF*, and *17βHSD2* expression and *SPP1* secretion). Adenomyosis organoids showed higher expression of TGF-β2 and SMAD3 and increased gene expression of *SPP1*, *PAEP*, *LIF*, and *17βHSD2* compared with control organoids. Our results demonstrate that organoids derived from endometria of adenomyosis patients and differentiated to secretory and gestational phases recapitulate native endometrial-tissue-specific features and disease-specific traits. Adenomyosis-derived organoids are a promising in vitro preclinical model to study impaired implantation and pregnancy disorders in adenomyosis and enable personalized drug screening.

## 1. Introduction

Adenomyosis is a benign uterine disease affecting 35% of women of reproductive age [1], characterized by invagination of endometrial glands and stroma into the myometrium [2]. Many mechanisms have been postulated to be involved in adenomyosis’ development and its associated symptoms, such as altered sex steroid signaling, excessive proliferation and invasiveness of the endometrium, and an abnormal immune response [3]. To date, the exact trigger of the disease is not known but there are two main hypotheses that attempt to explain adenomyosis origin [4,5]. The first and most commonly accepted theory lies in the invasiveness of the basal endometrium into the inner myometrium [6]. This is due to a combination of two events. On the one hand, endometrial epithelial cells undergo a process called epithelial–mesenchymal transition (EMT), in which they lose their cell polarity and intercellular junctions are disrupted, facilitating the transition to a mesenchymal state and increasing the invasive capacity [7,8]. On the other hand, there is a partial loss of continuity of the junctional zone (JZ) that facilitates the invasion and establishment of adenomyotic lesions [6]. The second theory is that adenomyotic lesions are generated de novo rather than originating from the eutopic endometrium [4,5]. This would be due to a differentiation of the misplaced Müllerian remnants into tissue that resembles endometrial tissue and would therefore grow in ectopic sites [9,10]. However, other authors support that ectopic lesions could be due to endometrial stem cells (ESCs) that are transported in retrograde menstruation [11,12], which have the ability to adhere, implant, differentiate, and propagate in ectopic locations [13]. The incidence and prevalence of adenomyosis is unknown due to the lack of an adequate and standardized diagnostic criteria [14,15]. The main diagnosis of adenomyosis is made either by imaging or by histological examination after hysterectomy, which implies the need to establish a diagnosis compatible with the woman’s reproductive intentions [16,17,18]. Moreover, no clear pattern of coexistence of adenomyosis with uterine diseases such as endometriosis, leiomyomas, and other uterine conditions has been established [18]. Although one third of women affected by adenomyosis is asymptomatic, most of them may present abnormal uterine bleeding, chronic pelvic pain, dysmenorrhea, or dyspareunia [19]. This condition can also result in infertility and miscarriages [19], leading most affected women to undergo assisted reproductive techniques [20]. Meta-analyses found that implantation, clinical and ongoing pregnancy, and live birth rates are significantly lower in patients with adenomyosis compared with healthy women, while miscarriage rates are higher [21,22,23].

Local inflammation [24], oxidative stress [25], impaired vascularization [26], hyperestrogenism [27], and epithelial–mesenchymal transition (EMT) [28] may be mechanisms involved in adenomyosis pathogenesis. Steroid hormones disruption, particularly hyperestrogenism, which ultimately results in progesterone resistance, are known to play a key role in its pathogenesis [3,27]. Since many of the mechanisms involved in the pathogenesis of adenomyosis are driven by estrogen upregulation, adenomyosis is widely known as an estrogen-dependent disease [27]. Estrogen induces proliferation of endometrial cells, which, in the case of adenomyotic endometrium, results in an overproliferation [29]. In this regard, it has been hypothesized that estrogen is responsible for triggering EMT in adenomyosis [30]. Transforming growth factor (TGF)-β1 and TGF-β2 are growth and differentiation factors involved in EMT induction and regulation [31], which are upregulated in secretory endometrium from patients with adenomyosis [32,33,34], suggesting dysfunctionality during the secretory phase. Further, TGF-β/SMAD (SMAD Family Member 3) signaling is implicated in adenomyosis’ pathology [35]. SMAD2/3 are downstream proteins of TGF-β pathway involved in endometrial function maintenance, including early glandular formation, appropriate endometrial hormonal response, and tumor suppression [36]. Specifically, it has been reported that SMAD3 is overexpressed in epithelial cells from eutopic adenomyosis endometrium in secretory phase [37]. Since SMAD3 is involved in endometrial receptivity and embryo implantation [38], these findings suggest SMAD3 as a key protein in adenomyosis-related infertility [37].

*SPP1* (Secreted Phosphoprotein 1) is an adhesion protein secreted by ECM (extracellular matrix) involved in endometrial-embryo signaling and embryo attachment [39], which is upregulated in the receptive phase human uterus [40]. *PAEP* (Progestagen Associated Endometrial Protein), also called Glycodelin-A (GdA), is a marker of morphological differentiation and an immunosuppressive molecule [41] secreted from luminal epithelial cells and localized in endometrial glands during pinopodes formation [42] in the secretory phase [43]. *PAEP* has been found to be elevated in the decidua throughout early pregnancy [44], being essential in the first processes of placentation [45]. *LIF* (Leukemia Inhibitory Factor) is a glycoprotein cytokine considered as an endometrial receptivity biomarker [46] that mediates implantation and immune response in several species [47], being crucial in decidualization regulation. *17βHSD2* (Hydroxysteroid 17-Beta Dehydrogenase) is an enzyme that metabolizes estradiol [48] and is overexpressed in both midgestation and placenta [49]. Furthermore, its activity is elevated during the secretory phase in diseased endometrium and estrogen metabolism is altered in the endometria of patients with estrogen-dependent benign diseases [50].

Patients with adenomyosis experience defective decidualization [51], altered uterine peristaltic activity [52] and endometrial receptivity [53], impaired embryo-maternal communication [54], and delayed pinopode formation, resulting in unsuccessful embryo implantation [55]. However, molecular mechanisms underlying these embryo implantation and pregnancy defects remain largely unknown, mainly due to the difficulty in obtaining secretory and gestational endometrium samples and the lack of reliable preclinical study models. Overcoming these challenges is crucial to significantly improve adenomyosis-related infertility therapies.

Organoids are self-organized in vitro in 3D structures that are genetically stable during long-term culture and viable after cryopreservation [56,57,58,59]. Organoids overcome the disadvantages of 2D culture by recapitulating the native microenvironment and reproducing native tissue characteristics [60]. Endometrial organoids may be differentiated, mimicking the midsecretory phase, in response to ovarian hormones estradiol (E2), progesterone (P4), and 8-Bromoadenosine3′,5′-cyclicmonophosphate (cAMP) [61,62] and acquire an early gestation phenotype with pregnancy hormones prolactin (PRL) and human placental lactogen (hPL) [61]. There are several patient-derived organoid models of endometrial diseases, such as endometriosis and endometrial cancers [63], which can be cryopreserved and used for preclinical studies [59].

Human organoids derived from adenomyosis eutopic endometrium and their differentiation to midsecretory and early pregnancy phases represent a powerful platform to study the dysregulated molecular mechanisms involved in implantation and pregnancy disorders. Generation of an organoid biobank representing healthy and pathological conditions would provide innovative and powerful preclinical study models for drug screening and personalized medicine.

## 2. Materials and Methods

### 2.1. Patient Samples

Endometrial biopsies were obtained from adenomyosis patients and healthy women (*n* = 6/group) at IVI Valencia. The study population underwent pelvic ultrasound examination as the routine workout in infertile women. All patients, cases, and controls were carefully scanned by transvaginal ultrasound. A heterogeneous myometrium with blurring of the endometrial border is the key for diagnosis of adenomyosis. In case of suspected adenomyosis, MRI (Magnetic Resonance Imaging) or hysteroscopic evaluation of the endometrial cavity was performed. Hysteroscopic findings were superficial openings on the endometrial cavity, endometrial hypervascularization, and cystic hemorrhagic lesions. Control group is based on young healthy women included in an egg donation (ED) program with a standard uterine volume, with no evidence of adenomyotic lesions by ultrasound, who were free from other gynecologic (endometrial or myometrial or ovarian) pathologies and without medication during the previous 3 months, as condition to be included in the ED program. Human tissue use was approved by the Clinical Ethics Committee at Hospital La Fe (2004-FIVI-039-HF; Valencia, Spain). Informed consent was provided.

### 2.2. Derivation and Culture of Organoids from Human Endometrial Tissue Samples

Endometrial biopsies from adenomyosis patients (*n* = 6) and healthy (*n* = 6) women were processed to isolate the epithelial glandular fractions [61]. Biopsies were mechanically and enzymatically digested with 50 U/mL Dispase II (Sigma-Aldrich, St. Louis, MO, USA, D4693) and 4 mg/mL Collagenase-V (Sigma-Aldrich, St. Louis, MO, USA, C9263). Supernatant was passed through 100 µm cell sieves (Corning, Tewksbury, MA, USA, 431752) to retain glandular elements; the pellet was resuspended in 15% DMEM/F12 (Invitrogen, Invitrogen, Paisley, UK, 12634010) and 85% Matrigel (Corning, Bedford, MA, USA, 354234) and 20 μL droplets were seeded. A total 250 μL of organoid Expansion Medium (ExM) (Table 1) were added and changed every 2 days. Organoid passage was made every 7 days, with pipetting up and down forcefully and centrifuging to disaggregate organoids and remove Matrigel. Pellets containing organoid fragments were resuspended in 15% DMEM/F12–85% Matrigel.

### 2.3. Differentiation of Endometrial Organoids to Secretory and Gestational Phases

Organoids in passage 3 (p3) were cultured with ExM for 7 days to maintain undifferentiated status (derived-organoids). For differentiation experiments [61], after 4 days in ExM, culture media was enriched with 10 nM E2 (Sigma-Aldrich, St. Louis, MO, USA, Sigma E4389) for 48 h; and with 10 nM E2, 1 µM P4 (Sigma-Aldrich, St. Louis, MO, USA, Sigma P7556), and 1 µM cAMP (Sigma-Aldrich, St. Louis, MO, USA, Sigma B7880) for 4 days to promote differentiation to the secretory phase (sec-organoids). To induce gestational phase (gest-organoids) differentiation, 20 ng/mL PRL (Peprotech, Cranbury, NJ, USA, 100-07) and 20 ng/mL hPL (R&D, Minneapolis, MN, USA, 5757-PL) were added to previously supplemented ExM for 8 days.

### 2.4. Immunohistochemical (IHC) and Immunofluorescence (IF) Characterization

To verify that Control- and adenomyosis (Adeno) derived-organoids reproduced biological and pathological features of the native tissue, organoids were fixed, paraffin embedded [64], and cut into 4-µm sections. Periodic acid-Schiff (PAS) (glycogen secretion) (Sigma-Aldrich, St. Louis, MO, USA, 395B) staining was performed according to manufacturer’s protocol. For assessment of MUC-1 (mucin-1) (glandular secretion), Ki67 (cell proliferation), SOX9 (SRY-Box Transcription Factor 9) (progenitor cells markers), TGF-β2, and SMAD3 (adenomyosis development) protein expression, samples were incubated with primary antibodies (Table 2) overnight at 4 °C. After endogenous peroxidase activity blockage, slides were incubated with labeled-polymer HRP (horseradish peroxidase), substrate-chromogen and counterstained with hematoxylin. To evaluate pan-cytokeratin (epithelial marker), vimentin (stromal component), laminin (basoapical polarity), and acetylated α-tubulin (cilia presence) expression, samples were incubated with primary antibodies overnight at 4 °C and their correspondent secondary antibody 45 min at RT (room temperature) (Table 2). Samples were visualized using a Nikon Eclipse 80i microscope. To measure SOX9, α-tubulin, TGF-β2, and SMAD3 protein expression levels, four images per sample were quantitatively assessed with Image ProPlus (Media Cybernetics, Rockville, MD, USA). Quantification was made by calculating the ratio between the area stained by the signal and the total area of the histological section.

### 2.5. Gene Expression Analysis

To confirm organoid differentiation into secretory and gestational phases, gene expression of implantation and placentation biomarkers *SPP1*, *PAEP*, *LIF*, and *17βHSD2* was evaluated by quantitative real time PCR (qRT-PCR) using a StepOnePlus system (Applied Biosystems, Waltham, MA, USA, 4376600). To evaluate the possible role of these biomarkers in implantation and pregnancy in adenomyosis, their expression was assessed in Control and Adeno differentiated organoids. Total RNA was extracted from organoids (*n* = 6/group) using Trizol reagent (Qiagen, Gilde, Germany, 79306). Gene expression levels were normalized with housekeeping gene *GAPDH* (Glyceraldehyde-3-Phosphate Dehydrogenase), quantified by the ΔΔCt method, and represented as fold-change in each group. Primers were designed using Primer Quest Tool (DNA Integrated Technologies) (Table 3).

### 2.6. ELISA

To assess proper sec-organoid differentiation, we evaluated *SPP1* protein secretion. Culture media from Adeno and Control derived- and sec-organoids were collected and supernatants were concentrated with Vivaspin2-concentrators (Generon, Slough, UK, VS0291). Osteopontin (*SPP1*) Human ELISA (Enzyme-Linked ImmunoSorbent Assay) (Invitrogen, Eugene, OR, USA, BMS2066) was performed in duplicate according to the manufacturer’s instructions.

### 2.7. Chromosomal Stability

Chromosomal stability of Control and Adeno derived-, sec-, and gest-organoids (*n* = 3/group) was evaluated using a Genome-Wide high-resolution Affymetrix-Cytoscan 750K-array (Affymetrix Inc, Santa Clara, CA, USA). DNA was extracted using the Cells and Tissue DNA-Isolation Micro-Kit (Norgen, Thorold, ON, Canada, 57300). Data were analyzed using Affymetrix Chromosome-Analysis Suite (ChAS4.2).

### 2.8. Statistical Analysis

Graphpad Prism 6.0 was used for statistical analyses. Two-tailed Student’s *t*-test and one-way ANOVA were used for comparisons between two and three groups, and *p* < 0.05 was considered statistically significant.

## 3. Results

### 3.1. Human Endometrial Organoids Can Be Derived from Adenomyosis Patients and Recapitulate Endometrial Gland Biology In Vivo

**Figure 1:**
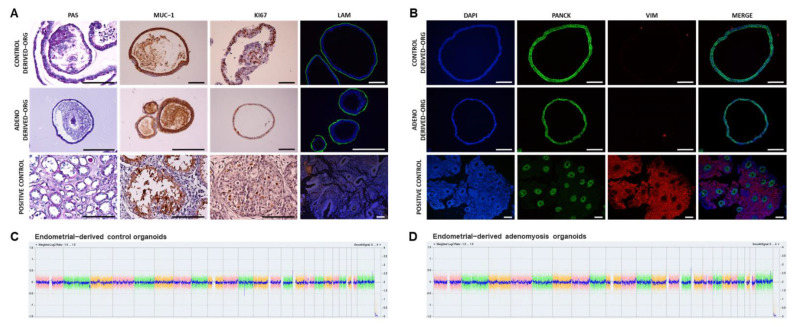
Characterization of glandular origin, proliferation, and epithelial polarity in human endometrial-derived organoids. Representative images of (**A**) PAS staining as well as MUC-1, Ki67, and laminin expression by IHC; (**B**) pan-cytokeratin and vimentin staining by IF; and chromosomal stability in Control (**C**) and Adeno (**D**) derived-organoids. Scale bars are 100 µm. Kidney, endometrium, and breast cancer samples were used as positive controls.

To evaluate if Adeno derived-organoids recapitulate the biological characteristics of the native endometrium, we determined their glandular epithelial origin, organoid structure, secretions, proliferation capacity, and apicobasal polarity maintenance (Figure 1A). Endometrial organoids were derived from healthy women for the control group. PAS staining confirmed organoid production of epithelial glycogen, a main component of endometrial glandular secretions [61]. MUC-1 was secreted by organoids into the luminal compartment, as observed in human endometrial gland lumen. Ki67 expression in the organoids demonstrated maintenance of proliferative capacity. Laminin presence along the basolateral membrane confirmed that organoid epithelial cells maintain apicobasal polarity.

Pan-cytokeratin (glands) and vimentin (stroma) expression confirmed correct isolation of the epithelial glands (Figure 1B). Pan-cytokeratin was expressed in organoid cell cytoplasmic compartment while vimentin was not expressed, corroborating the epithelial origin of the organoids.

### 3.2. Differentiation to Secretory and Gestational Phases of Human Adenomyosis-Derived Organoids in Response to Hormonal Treatments

To reproduce secretory (sec) and gestational (gest) in vivo conditions, human-derived organoids were exposed to E2, P4, and cAMP to promote transition to receptive state, and to PRL and hPL to mimic the early gestational phase. We confirmed that glandular epithelial origin was preserved after differentiation in Control and Adeno sec- and gest-organoids (Supplementary Figure S1A,B).

**Figure 2:**
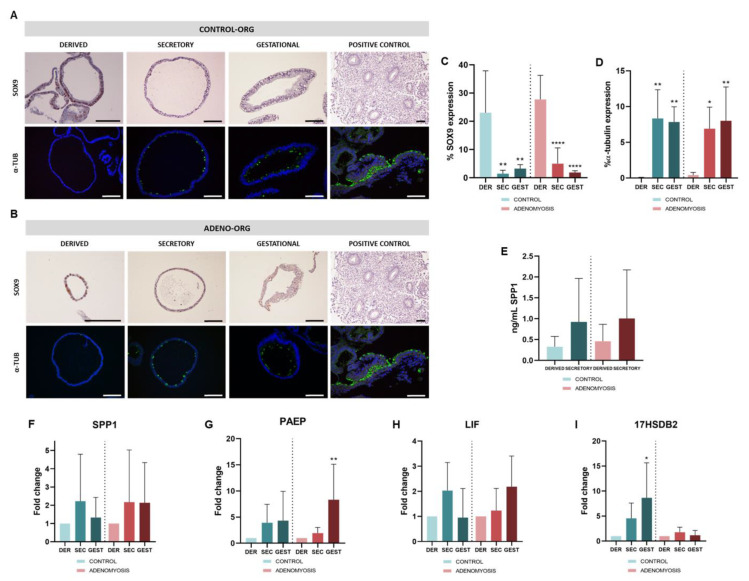
Characterization of sec-organoids and gest-organoids. Representative images of SOX9 and α-tubulin expression in sec-organoids and gest-organoids derived from (**A**) Control and (**B**) Adenomyosis patient samples by IHC and IF. Quantification of (**C**) SOX9 and (**D**) α-tubulin protein expression in derived, secretory, and gestational Control and Adeno organoids. (**E**) *SPP1* protein secretion levels in derived, secretory, and gestational Control and Adeno organoids by ELISA. (**F**) *SPP1*, (**G**) *PAEP*, (**H**) *LIF*, and (**I**) *17βHSD2* gene expression in derived, secretory, and gestational Control and Adeno organoids by qRT-PCR. Scale bars are 100 µm. Endometrium was used as a positive control. * *p* < 0.05, ** *p* < 0.01, **** *p* < 0.0001.

SOX9 and α-tubulin expression was evaluated by IHC and IF to confirm differentiation of Control (Figure 2A) and Adeno (Figure 2B) sec- and gest-organoids. SOX9, a progenitor cell marker, was increased in derived organoids and, after differentiation, its expression was significantly reduced in Control (*p* = 0.0012 and 0.0025) and Adeno (*p* < 0.0001 for both) sec- and gest-organoids (Figure 2C), as occurs in decidual glands in vivo. Secretory- and gestational-phase hormonal treatment significantly promoted formation of ciliated cells, indicated by expression of acetylated α-tubulin, in Control (*p* = 0.0031 and 0.0048) and Adeno (*p* = 0.0146 and 0.0056) sec- and gest-organoids (Figure 2D), as occurs in vivo. Differentiation was evaluated at the protein level by ELISA of implantation marker *SPP1* secretion into the culture media (Figure 2E). Secreted *SPP1* levels were higher in Control and Adeno sec-organoids compared with derived organoids, confirming differentiation.

Higher expression of the secretory and gestational markers *SPP1*, *PAEP*, *LIF*, and *17βHSD2* in sec- and gest-organoids compared with derived-organoids from Control and Adeno corroborated the differentiation to secretory and gestational phase in both conditions (Figure 2F–I). Hormonal treatment to induce secretory and gestational phases in Control organoids increased expression of *SPP1* (Fold Change (FC) = 2.215 ± 2.577; FC = 1.330 ± 1.102), *PAEP* (FC = 3.926 ± 3.535; FC = 4.331 ± 5.599), *LIF* (FC = 2.031 ± 1.116; FC = 0.9502 ± 1.161), and *17βHSD2* (FC = 4.520 ± 3.080; FC = 8.641 ± 7.006, *p* = 0.0168) compared with Control untreated, derived organoids. In sec- and gest-phase Adeno organoids, expression of *SPP1* (FC = 2.172 ± 2.853; FC = 2.137 ± 2.195), *PAEP* (FC = 1.948 ± 1.0.72; FC = 8.330 ± 6.775, *p* = 0.0145), *LIF* (FC = 1.234 ± 0.8801; FC = 2.187 ± 1.218), and *17βHSD2* (FC = 1.760 ± 1.021; FC = 1.163 ± 0.9616) were also increased compared with Adeno untreated, derived organoids.

### 3.3. Human Adenomyosis-Derived Organoids Maintain Chromosomal Stability after Differentiation

Chromosomal stability of Control (*n* = 3) and Adeno (*n* = 3) derived-organoids (Figure 1C,D), sec-organoids, and gest-organoids (Supplementary Figure S1C,D) were assessed using a cytogenetics microarray and compared against a reference genome. No DNA copy number alterations were observed after derived-organoids culture and passage until p3. Exposure to secretory and gestational phase hormonal treatment had no effect on chromosomal stability. All established organoid lines from women with adenomyosis and controls showed a normal 46, XX karyotype.

### 3.4. Human Adenomyosis Secretory and Gestational Organoids Recapitulate Disease-Specific Traits

**Figure 3:**
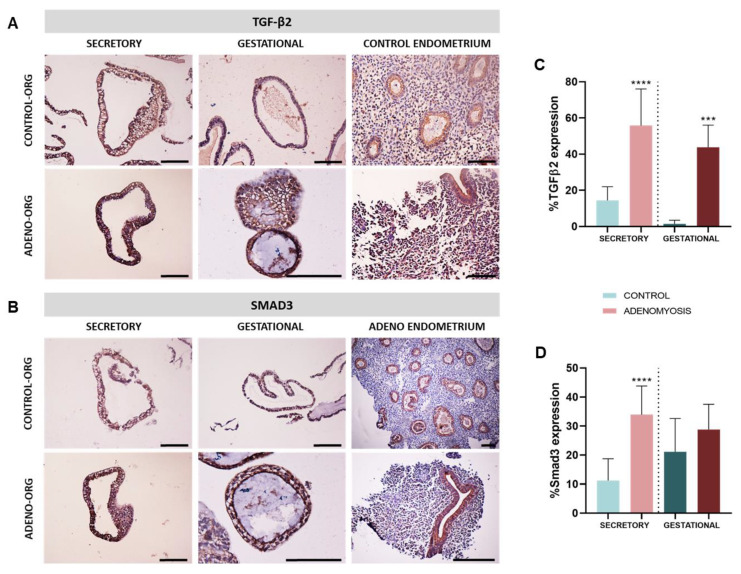
Pathological characterization of adenomyosis secretory and gestational organoids. Representative images of (**A**) TGF-β2 and (**B**) SMAD3 in Adeno and Control sec-organoids and gest-organoids by IHC. Quantification of (**C**) TGF-β2 and (**D**) SMAD3 protein expression in Adeno and Control sec-organoids and gest-organoids. *** *p* < 0.001, **** *p* < 0.0001.

To determine whether organoids are a suitable in vitro model of the in vivo pathological features of adenomyosis, TGF-β2 and SMAD3 expression were evaluated by IHC in Control and Adeno (*n* = 6/group) sec- and gest-organoids. TGF-β2 and SMAD3 (Figure 3A,B) expression were upregulated in Adeno compared with Control sec-organoids (TGF-β2: 55.78 ± 20.26% vs. 14.45 ± 7.51%, *p* < 0.0001 and SMAD3: 33.95 ± 9.88% vs. 11.22 ± 7.51%, *p* < 0.0001), and gest-organoids (TGF-β2: 43.81 ± 12.22% vs. 1.41 ± 2.00%, *p* = 0.0003 and SMAD3: 28.81 ± 87.69% vs. 21.16 ± 11.44%, *p* = 0.3282) (Figure 3C,D), as observed in adenomyosis [32,37].

### 3.5. Dysregulation of Secretory and Gestational Biomarkers in Human Adenomyosis Organoids

**Figure 4:**
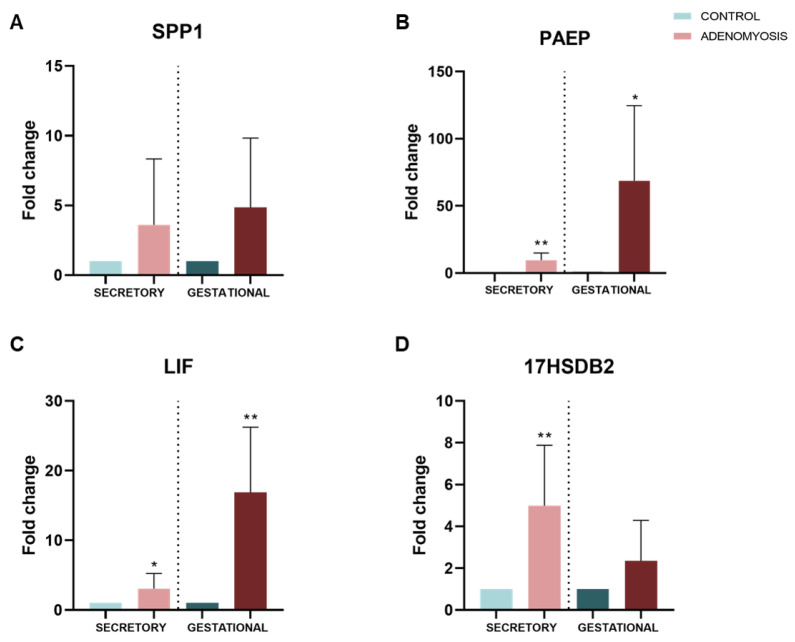
Gene expression of secretory and gestational endometrial biomarkers involved in regulation of implantation and placentation. (**A**) *SPP1*, (**B**) *PAEP*, (**C**) *LIF*, and (**D**) *17βHSD2* gene expression in Adeno and Control secretory and gestational organoids by qRT-PCR. Scale bars are 100 µm. Endometrium was used as a positive control. * *p* < 0.05, ** *p* < 0.01.

As a first approach in understanding impaired implantation and pregnancy disorders characteristic of women with adenomyosis, expressions of secretory and gestational endometrial biomarkers *SPP1*, *PAEP*, *LIF*, and *17βHSD2* were evaluated in adenomyosis sec- and gest-organoids by qRT-PCR and compared with Control sec- and gest-organoids (Figure 4A–D). These biomarkers are involved in regulation of implantation (midsecretory phase) and placentation (early pregnancy); their expression was increased in Adeno sec- and gest-organoids compared with Control organoids in the same phases ((*SPP1* FC = 3.603 ± 4.733, *p* = 0.3636; FC = 4.850 ± 4.983, *p* = 0.0879), (*PAEP* FC = 9.610 ± 5.292, *p* = 0.0030; FC = 68.70 ± 55.88, *p* = 0.0141); (*LIF* FC = 3.054 ± 2.179, *p* = 0.0436; FC = 16.85 ± 9.388, *p* = 0.0020); *17βHSD2* FC = 4.984 ± 2.892, *p* = 0.0071; FC = 2.345 ± 1.939, *p* = 0.1201)).

## 4. Discussion

Adenomyosis is one of the most widespread uterine conditions among women of reproductive age, but so far, there have been no robust in vitro models that reproduce its pathological features to study the molecular mechanisms involved in its pathogenesis and infertility disorders. We have been able to develop a human organoid model of the adenomyosis secretory and gestational endometrium, recapitulating specific native tissue features and disease traits. These organoids will provide powerful preclinical research models to study adenomyosis-impaired implantation and increased miscarriages as well as to enable personalized medicine.

Previous studies in the field of reproductive medicine have relied on organoids such as 3D in vitro models to study endometrial physiology and disease [60,63,65,66,67]. In this regard, organoids have been exploited in the study of defective endometrial proliferation, such as endometriosis or endometrial cancer [63,68], disorders affecting decidualization [67], endocrine disruptors [67], or gynecological infections [69,70]. In addition, its potential in personalized medicine or as a source of biological material in regenerative therapy is becoming increasingly evident [71,72,73].

We derived endometrial organoids from healthy women, as previously described [61,64], and for the first time, have reproducibly established organoids from endometria of adenomyosis patients. Organoids recapitulated the molecular signatures of in vivo endometrial glands. Histology confirmed expression of several cytokeratins in Control and Adeno organoids, which exert structural function in epithelial cells and have an important role in differentiation and tissue function [74]. Likewise, glycogen (glandular secretions) and MUC-1 (mucus release) presence in the luminal compartments of Adeno organoids suggests that they mimic glandular tissue functioning in the same way as Control organoids. Lastly, both Control and Adeno organoids maintained proliferative capacity (Ki67), cell apicobasal polarity (laminin), and chromosomal stability after successive passages.

Hormone responsiveness of healthy endometrial organoids has been reported [75,76,77]; so, we wanted to evaluate this response ability in Adeno organoids towards the differentiation into secretory and gestational endometrium. Similar to our Control organoids, Adeno organoids showed E2 and P4 treatment sensitivity, acquiring a secretory phenotype, and when further stimulated with pregnancy (hPL) and stroma (PRL) signals, adopted gestational endometrium characteristics. This was substantiated by decreased expression of progenitor cell marker SOX9, indicating differentiation processes, appearance of ciliated cells (α-tubulin), and increased synthesis of *SPP1*. Acquisition of differentiated phenotypes was further verified by upregulation of *SPP1*, *PAEP*, *LIF*, and *17βHSD2*, which are expressed by secretory endometrium and decidua. *SPP1* levels are high in the human uterus in the receptive phase [40] and luminal epithelium in early pregnancy in pigs [75], suggesting that *SPP1* is essential for endometrial receptivity and implantation [78]. *PAEP* secretion is increased by P4 midpregnancy [79,80], relating it to endometrial receptivity and early pregnancy. Reported increased *LIF* expression in mouse endometrium during late diestrus phase, and in the human endometrium during the secretory phase and mid- to late-pregnancy [47,81,82], suggest that *LIF* is an endometrial receptivity biomarker. High *17βHSD2* expression and activity is found in secretory phase, midgestation, and term human placentas [49].

Adenomyosis is thought to be promoted by EMT, which is induced and regulated by factors including TGF-β1 and TGF-β2 [31,34]. The TGF-β/SMAD3 pathway participates in embryo implantation, as TGF-βs and SMADs largely expressed in human endometrium during implantation window [38]. TGF-β/SMAD3 signaling is a major mechanism involved in endometrial fibrosis [37] and plays a key role in adenomyosis development [35]. Further, patients with adenomyosis present increased TGF-β2 and SMAD3 levels in their eutopic endometrium during the secretory phase compared with disease-free women [32,33,37]] SMAD3. Remarkably, our secretory adenomyosis organoids showed significant TGF-β2 and SMAD3 upregulation compared with controls, confirming successful secretory-phase differentiation and accurate reproduction of specific disease traits. Thus, this adenomyosis in vitro model appears suitable for studying patients with impaired implantation.

Adenomyosis causes defective placentation [83], which is significantly associated with increased risk of preeclampsia [84,85]. TGF-βs—through activation of downstream signaling mediators—and SMAD2/3 are triggering factors for preeclampsia, resulting in abnormal placental development [86,87]. SMAD3 is further involved in key gestational processes, immune regulation, and inflammation and its altered expression may be associated with recurrent pregnancy loss [88] and preterm birth [89]. Accordingly, our gestational adenomyosis organoids (imitating early pregnancy) showed increased levels of TGF-β2 and SMAD3, recapitulating adenomyosis tissue characteristics. This implies that gestational differentiated organoids represent a potent preclinical platform and research approach for studying placentation and early-pregnancy disorders in women with adenomyosis.

*SPP1*, *PAEP*, *LIF*, and *17βHSD2* expression were upregulated in adenomyosis sec- and gest-organoids compared with control organoids, indicating possible molecular mechanisms involved in adenomyosis-impaired implantation and pregnancy disorders. *SPP1*, which is involved in endometrial-embryo signaling and embryo attachment [39], was upregulated in adenomyosis secretory organoids compared with healthy organoids, as it was described in adenomyosis women ectopic endometrium [90]. Increased *SPP1* in our gestational adenomyosis organoids compared with control suggests abnormal endometrial *SPP1* expression during implantation window [90] and placentation [91] and could contribute to adenomyosis-related infertility. *PAEP*, a morphological differentiation marker and immunosuppressive molecule [41] secreted from luminal epithelial cells and localized in endometrial glands during pinopode formation [42], was significantly upregulated in our Adeno secretory organoids. Abnormal *PAEP* expression during the secretory phase in endometriosis eutopic endometrium [43] suggests that dysregulated *PAEP* expression could be related to impaired endometrial receptivity. *PAEP* was also increased in Adeno gestational organoids compared with healthy organoids. This marker is abundant in the decidua during early pregnancy and is crucial in placentation events and fetomaternal defense, regulating trophoblast and immune cell functions during early pregnancy [45]. Thus, its upregulation in adenomyosis sec- and gest-organoids suggests that abnormal endometrial *PAEP* levels could be involved in early pregnancy loss, preeclampsia, and recurrent miscarriage in women with adenomyosis, as previously suggested for endometriosis disease [43].

Our data showed significant upregulation of *LIF*—a glycoprotein cytokine involved in decidualization and immune response [47]—in secretory and gestational adenomyosis organoids compared with control. These results are not in line with previous works reporting lower *LIF* levels in adenomyosis patients’ endometrium, but the critical point is that the control group in these studies included women with other gynecological disorders [92,93]. As *LIF* regulates the Wnt/β-catenin pathway, which is involved in uterine preparation for implantation and EMT regulation [94], altered expression of this marker may be related to impaired implantation and altered EMT, possibly driving endometrial gland and stroma invagination into the myometrium, characteristic of adenomyosis [28].

Finally, *17βHSD2* is altered in the eutopic endometrium of adenomyosis, endometriosis, and leiomyoma patients [95,96], leading to estrogen metabolism alteration in estrogen-dependent benign disease patient endometria [50]. The observed *17βHSD2* upregulation in secretory adenomyosis organoids aligns with previous studies demonstrating that *17βHSD2* activity is increased in the endometrial secretory phase in diseased but not in disease-free endometrium [50]. In mice, *17βHSD2* disruption results in placentation defects and embryonic lethality [97]. Thus, the observed increase in *17βHSD2* expression in Adeno gestational organoids in the present study suggests a relationship between *17βHSD2* dysregulation in the endometrial gestational phase and associated early-pregnancy alterations in women with adenomyosis. However, further studies are needed in order to determine more accurately the involvement of these genes, as well as to describe other potential genes implicated in implantation and early pregnancy disorders in patients with adenomyosis.

The endometrial organoid model does not include stromal cells, which are involved in decidualization and other important processes, implying a lack of epithelial–stromal paracrine and autocrine crosstalk [67,98]. Regarding microenvironment communication, Matrigel does not allow us to reliably simulate tissue-specific cell–ECM interactions, with its replacement by decellularized endometrial hydrogels being a potential alternative [99]. In addition, adenomyosis organoid model only reproduces the endometrial component, leaving unstudied all the mechanisms involved in the damage that occurs in the JZ and myometrium [6]. However, in recent years, these limitations are being addressed; for example, organoid implantation models have been implemented in such a way that would allow accessibility to the luminal compartment [98,100]. Beyond that, our adenomyosis organoid model opens insights to the development of microfluidic devices and sensor systems that would help to optimize and standardize organoid cultures [101], solving then some of the mentioned limitations and improving the study of this condition.

Many authors have discussed the possibility that adenomyosis and endometriosis have a common origin and are therefore different manifestations of the same disease [102,103,104]. Since endometriosis organoids models have been already established [63] but to date there is, to our knowledge, no model of adenomyosis disease, this study could provide a new insight into the possible common mechanisms involved in the development of both diseases, as well as the associated infertility.

In conclusion, our adenomyosis organoid model maintains biological and pathological characteristics observed in secretory and gestational adenomyosis patients’ eutopic endometria. This model provides new knowledge about the possible role of implantation and early gestational biomarkers in adenomyosis-related infertility, opening avenues for further studies of these biomarkers and for development of therapeutic options for personalized treatments.

## 5. Conclusions

Here, we have successfully derived organoids from adenomyosis patients for the first time. Patient-derived adenomyosis organoids can be established and cryopreserved, allowing generation of a patient-specific biobank that would permit their use as a preclinical model for drug screening and promoting development of personalized medicine to improve implantation and avoid pregnancy disorders in adenomyosis patients. This is the first model demonstrating recapitulation of adenomyosis tissue origin characteristics at molecular and histological levels, which entails a step forward in generation of robust preclinical models that faithfully mimic this human endometrial pathology.

## Figures and Tables

**Table 1:** Expansion medium (ExM) composition.

Product	Company	Product Number	Concentration in ExM
Advance DMEM/F12	Life Technologies	12634010	1X
N2 supplement	Life Technologies	17502048	1X
B27 supplement minus vitamin A	Life Technologies	12587010	1X
Primocin	Invivogen	ant-pm-1	100 μg/ml
N-Acetyl-L-cysteine	Sigma	A9165	1.25 mM
L-glutamine	Sigma	G7513	2 mM
Recombinant human EGF	Peprotech	AF-100-15	50 ng/ml
Recombinant human Noggin	Peprotech	120-10c	100 ng/ml
Recombinant human Rspondin-1	Peprotech	120-38	500 ng/ml
Recombinant human FGF-10	Peprotech	100-26	100 ng/ml
Recombinant human HGF	Peprotech	100-39	50 ng/ml
ALK-4, -5, -7 inhibitor, A83-01	Peprotech	9094360	500 nM
Nicotinamide	Sigma	N0636	10 nM

Abbreviations: DMEM/F12 (Dulbecco’s Modified Eagle Medium: Nutrient Mixture F-12), EGF (Epidermal Growth Factor), FGF-10 (Fibroblast Growth Factor 10), HGF (Hepatocyte Growth Factor), ALK (Activin Receptor-like Kinase).

**Table 2:** Primary and secondary antibodies.

Antibody	Company	Product Number	Concentration
Anti-MUC-1	Abcam	ab109185	1:250
Anti-SOX9	Abcam	ab185966	1:100
Anti-Ki67	Dako	M7240	1:100
Anti-TGF-β2	Abcam	ab36495	1:1000
Anti-Smad3	Abcam	ab40854	1:500
Anti-PanCK	Abcam	ab86734	1:100
Anti-Vimentin	Abcam	ab92547	1:250
Anti-Laminin	Abcam	ab11575	1:200
Anti-acetylated α-tubulin	Santa Cruz Bt	611B1	1:500
AlexaFluor 488 goat antimouse IgG1	Invitrogen	A21121	1:500
AlexaFluor 555 goat antirabbit IgG	Invitrogen	A21429	1:500
AlexaFluor 488 goat antirabbit IgG	Invitrogen	A11034	1:500
AlexaFluor 488 goat antimouse IgG	Invitrogen	A11029	1:500

Abbreviations: MUC-1 (mucin-1), SOX9 (SRY-Box Transcription Factor 9), TGF Transforming growth factor), Smad3 (SMAD Family member 3), PanCK (Pancytokeratin), IgG (Immunoglobulin G).

**Table 3:** Primers sequences.

GENE	Forward Sequence	Reverse Sequence
*SPP1*	CGAGGTGATAGTGTGGTTTATG	GTCTGTAGCATCAGGGTACT
*PAEP*	ATGGCGACCAACAACATC	CTCTCCAAGGACCTTCTTCT
*LIF*	AACTGGCACAGCTCAATG	ATAGCTTGTCCAGGTTGTTG
*17HSDß2*	TGAATGTCAGCAGCATGG	GGAAAGCTCCAGTCTCATAAC
*GAPDH*	AACGTGTCAGTGGTGGACCTGA	ACCACCCTGTTGCTGTAGCCAA

Abbreviations: *SPP1* (Secreted Phosphoprotein 1), *PAEP* (Progestagen Associated Endometrial Protein), *LIF* (Leukemia Inhibitory Factor), *17HSDβ2* (Hydroxysteroid 17-Beta Dehydrogenase), *GAPDH* (Glyceraldehyde-3-Phosphate Dehydrogenase).

## Data Availability

The data presented in this study are available on request from the corresponding author.

## Supplementary Materials

The following supporting information can be downloaded at: https://www.mdpi.com/article/10.3390/jpm12020219/s1, Figure S1: Characterization of glandular origin, proliferation, and epithelial polarity in human secretory and gestational organoids; Table S1: Expansion medium (ExM) composition; Table S2: Primary and secondary antibodies; Table S3: Primers sequences.
