# Supplementary material for: Establishment of Adenomyosis Organoids as a Preclinical Model to Study Infertility
Source: J Pers Med. 2022 Feb 4;12(2):219. doi: 10.3390/jpm12020219 (PMC8876865; doi:10.3390/jpm12020219)
Supplement: Supplementary file 1 [file jpm-12-00219-s001.zip › jpm-1546449 Figure S1.pdf]

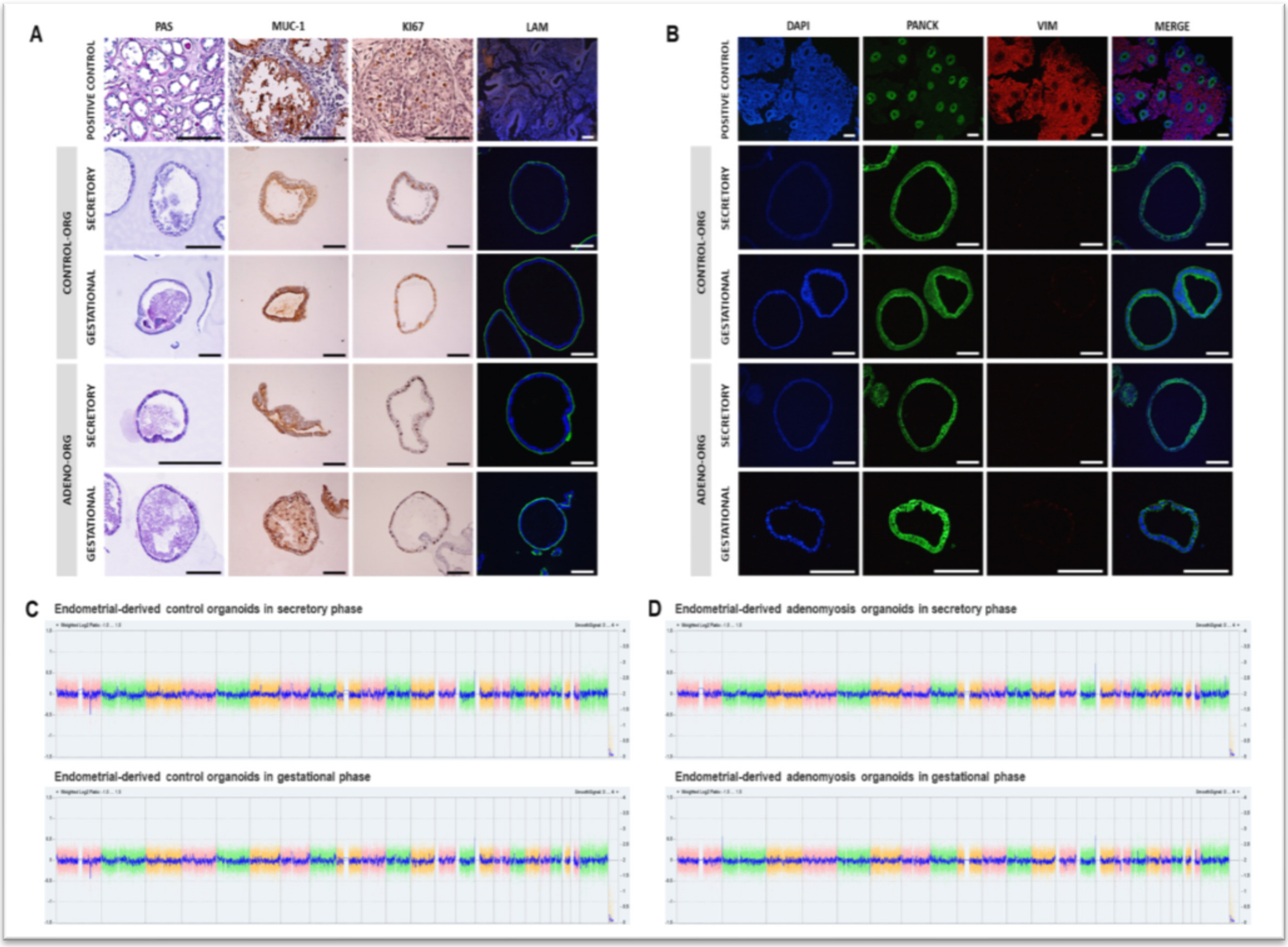

Figure S1. Characterization of glandular origin, proliferation, and epithelial polarity in human secretory and gestational organoids. Representative images of (A) PAS staining as well as MUC-1, Ki67, and laminin expression by IHC, (B) pan-cytokeratin and vimentin staining by IF. Chromosomal stability in secretory and gestational Control (C) and Adeno (D) organoids. Scale bars are 100 µm. Kidney, endometrium, and breast cancer samples were used as positive controls.
