# Supplementary material for: Establishment of Adenomyosis Organoids as a Preclinical Model to Study Infertility
Source: J Pers Med. 2022 Feb 4;12(2):219. doi: 10.3390/jpm12020219 (PMC8876865; doi:10.3390/jpm12020219)
Supplement: Supplementary file 1 [file jpm-12-00219-s001.zip › Supplementary Table S1.pdf]

**Supplementary Table S1. Expansion medium (ExM) composition**

| Product | Company | Product number | Concentration in ExM |
|---|---|---|---|
| **Advance DMEM/F12** | Life Technologies | 12634010 | 1X |
| **N2 supplement** | Life Technologies | 17502048 | 1X |
| **B27 supplement minus vitamin A** | Life Technologies | 12587010 | 1X |
| **Primocin** | Invivogen | ant-pm-1 | 100 µg/ml |
| **N-Acetyl-L-cysteine** | Sigma | A9165 | 1.25 mM |
| **L-glutamine** | Sigma | G7513 | 2 mM |
| **Recombinant human EGF** | Peprotech | AF-100-15 | 50 ng/ml |
| **Recombinant human Noggin** | Peprotech | 120-10c | 100 ng/ml |
| **Recombinant human Rspondin-1** | Peprotech | 120-38 | 500 ng/ml |
| **Recombinant human FGF-10** | Peprotech | 100-26 | 100 ng/ml |
| **Recombinant human HGF** | Peprotech | 100-39 | 50 ng/ml |
| **ALK-4, -5, -7 inhibitor, A83-01** | Peprotech | 9094360 | 500 nM |
| **Nicotinamide** | Sigma | N0636 | 10 nM |
