# Supplementary material for: Establishment of Adenomyosis Organoids as a Preclinical Model to Study Infertility
Source: J Pers Med. 2022 Feb 4;12(2):219. doi: 10.3390/jpm12020219 (PMC8876865; doi:10.3390/jpm12020219)
Supplement: Supplementary file 1 [file jpm-12-00219-s001.zip › Supplementary Table S2.pdf]

Supplementary Table S2. Primary and secondary antibodies

Antibody	Company	Product number	Concentration
**Anti-MUC-1**	Abcam	ab109185	1:250
**Anti-SOX9**	Abcam	ab185966	1:100
**Anti-Ki67**	Dako	M7240	1:100
**Anti-TGF-β2**	Abcam	ab36495	1:1000
**Anti-Smad3**	Abcam	ab40854	1:500
**Anti-PanCK**	Abcam	ab86734	1:100
**Anti-Vimentin**	Abcam	ab92547	1:250
**Anti-Laminin**	Abcam	ab11575	1:200
**Anti-acetylated α-tubulin**	Santa Cruz Bt	611B1	1:500
**AlexaFluor 488 goat anti-mouse IgG1**	Invitrogen	A21121	1:500
**AlexaFluor 555 goat-anti-rabbit IgG**	Invitrogen	A21429	1:500
**AlexaFluor 488 goat anti-rabbit IgG**	Invitrogen	A11034	1:500
**AlexaFluor 488 goat anti-mouse IgG**	Invitrogen	A11029	1:500
