# Supplementary material for: Establishment of Adenomyosis Organoids as a Preclinical Model to Study Infertility
Source: J Pers Med. 2022 Feb 4;12(2):219. doi: 10.3390/jpm12020219 (PMC8876865; doi:10.3390/jpm12020219)
Supplement: Supplementary file 1 [file jpm-12-00219-s001.zip › Supplementary Table S3.pdf]

Supplementary Table S3. Primers sequences

GENE	Forward sequence	Reverse sequence
SPP1	CGAGGTGATAGTGTGGTTTATG	GTCTGTAGCATCAGGGTACT
PAEP	ATGGCGACCAACAACATC	CTCTCCAAGGACCTTCTTCT
LIF	AACTGGCACAGCTCAATG	ATAGCTTGTCCAGGTTGTTG
17HSDβ2	TGAATGTCAGCAGCATGG	GGAAAGCTCCAGTCTCATAAC
GAPDH	AACGTGTCAGTGGTGGACCTGA	ACCACCCTGTTGCTGTAGCCAA
